# Carriage of distinct *bla*_KPC-2_ and *bla*_OXA-48_ plasmids in a single ST11 hypervirulent *Klebsiella pneumoniae* isolate in Egypt

**DOI:** 10.1186/s12864-021-08214-9

**Published:** 2022-01-08

**Authors:** Yanxian Yang, Yongqiang Yang, Mohamed Abd El-Gawad El-Sayed Ahmed, Mingyang Qin, Ruowen He, Yiping Wu, Xiaoxue Liang, Lan-Lan Zhong, Ping Chen, Baoguo Deng, Reem Mostafa Hassan, Weihong Wen, Lingqing Xu, Xubin Huang, Lin Xu, Guo-Bao Tian

**Affiliations:** 1 Department of Microbiology, Zhongshan School of Medicine, Sun Yat-sen University, 74 Zhongshan 2nd Road, Guangzhou, 510080 China; 2 Key Laboratory of Tropical Diseases Control (Sun Yat-sen University), Ministry of Education, Guangzhou, 510080 China; 3 School of Pharmaceutical Sciences (Shenzhen), Sun Yat-sen University, Guangzhou, 510006 China; 4 Department of Microbiology and Immunology, Faculty of Pharmaceutical Sciences and Drug Manufacturing, Misr University for Science and Technology, Cairo, 6th of October City, Egypt; 5 Department of Pathogen Biology, School of Basic Medical, Xinxiang Medical University, Xinxiang, 453003 China; 6 School of Laboratory Medicine, Chengdu Medical College, Chengdu, 610500 China; 7 Department of Clinical and Chemical Pathology, Faculty of Medicine, Cairo University, Cairo, Egypt; 8 Department of Clinical Laboratory, The Sixth Affiliated Hospital of Guangzhou Medical University, Qingyuan People’s Hospital, Qingyuan, 511518 China; 9 Department of Pulmonary and Critical Care Medicine, the First Affiliated Hospital, Sun Yat-sen University, Guangzhou, China; 10 Research Center for Clinical Laboratory Standard, Zhongshan School of Medicine, Sun Yat⁃sen University, Guangzhou, China; 11 School of Medicine, Xizang Minzu University, Xianyang, 712082 Shaanxi China

**Keywords:** *K. pneumoniae*, KPC-2, OXA-48, Hypervirulence, Egypt

## Abstract

**Background:**

Carbapenem-resistant hypervirulent *K. pneumoniae* (CR-hvKP) causes serious infections with significant morbidity and mortality. However, the epidemiology and transmission mechanisms of CR-hvKP and the corresponding carbapenem-resistant plasmids require further investigation. Herein, we have characterized an ST11 *K. pneumoniae* strain EBSI041 from the blood sample encoding both hypervirulence and carbapenem resistance phenotypes from a patient in Egypt.

**Results:**

*K. pneumoniae* strain EBSI041 showed multidrug-resistance phenotypes, where it was highly resistant to almost all tested antibiotics including carbapenems. And hypervirulence phenotypes of EBSI041 was confirmed by the model of *Galleria mellonella* infection. Whole-genome sequencing analysis showed that the hybrid plasmid pEBSI041-1 carried a set of virulence factors *rmpA*, *rmpA2*, *iucABCD* and *iutA*, and six resistance genes *aph(3′)-VI*, *armA*, *msr(E)*, *mph(E), qnrS*, and *sul2*. Besides, *bla*_OXA-48_ and *bla*_SHV-12_ were harboured in a novel conjugative IncL-type plasmid pEBSI041-2. The *bla*_KPC-2_-carrying plasmid pEBSI041-3, a non-conjugative plasmid lacking the conjugative transfer genes, could be transferred with the help of pEBSI041-2, and the two plasmids could fuse into a new plasmid during co-transfer. Moreover, the emergence of the p16HN-263_KPC-like plasmids is likely due to the integration of pEBSI041-3 and pEBSI041-4 via IS*26*-mediated rearrangement.

**Conclusion:**

To the best of our knowledge, this is the first report on the complete genome sequence of KPC-2- and OXA-48-coproducing hypervirulent *K. pneumoniae* from Egypt. These results give new insights into the adaptation and evolution of *K. pneumoniae* during nosocomial infections.

**Supplementary Information:**

The online version contains supplementary material available at 10.1186/s12864-021-08214-9.

## Background

Carbapenem-resistant *Klebsiella pneumoniae* (CRKP) is one of the most critical threats to global public health associated with significant morbidity and mortality [1–3]. *K. pneumoniae* producing KPCs, NDMs, and OXA-48-like carbapenemases have become rapidly disseminated worldwide [2]. The *bla*_KPC_ and *bla*_NDM_ genes in *K. pneumoniae* have been reported on multiple plasmid types, including IncF, IncA/C, IncR, IncX and IncL/M. Among them, the IncF and IncA/C type plasmids are predominantly responsible for the transfer of *bla*_KPC_ and *bla*_NDM_, respectively [4, 5]. Besides, the *bla*_KPC_ gene is strongly associated with Tn*4401* flanking by IS*Kpn6* and IS*Kpn7*, and the *bla*_NDM_ gene is closely related to Tn*125* structure with two IS*Aba125* elements [6, 7]. Unlike KPCs and NDMs, IncL group plasmid has been shown to be the major genetic carrier for *bla*_OXA_ in *K. pneumoniae* and the composite transposon Tn*1999* is mainly responsible for integration of the *bla*_OXA_ gene [4]. Furthermore, dissemination of KPC-producing *K. pneumoniae* in worldwide is largely caused by expansion of the dominant ST258 clones [8]. Differently, the *bla*_OXA_ and *bla*_NDM_ genes are detected in various *K. pneumoniae* clones, in which ST11 is a major high-risk sequence type in many countries, such as China [9, 10], Australia [11], Poland [12], Spain [13] and Turkey [14]. Co-carriage of different carbapenems resistance genes is not common but renders clinical *K. pneumoniae* strains extremely highly resistant to different carbapenems, which leads to more difficult infection treatment [15, 16].

Seriously, carbapenem-resistant hypervirulent *K. pneumoniae* (CR-hvKP) has been increasingly reported in nosocomial infection and can cause higher mortality [17, 18]. The plasmid-mediated genetic factors conferring the hypervirulent phenotype including *rmpA* and *rmpA2* (regulators that increase capsule production), and several siderophore gene clusters [19]. Notably, a multinational prospective cohort study warns of the severity of carbapenem resistance in low-income and middle-income countries, including Egypt [3]. In the previous studies, the high prevalence of carbapenemase-mediated resistance in *K. pneumoniae* isolates in the clinical setting from Egypt was reported [20–23], but few studies have analyzed the genome characteristic of carbapenem-resistant *K. pneumoniae* by whole-genome sequencing. In this study, we report the in-depth characterization of co-producing *bla*_KPC-2_ and *bla*_OXA-48_ hypervirulent ST11 *K. pneumoniae* strain from Egypt. The dissemination of two plasmids carrying *bla*_OXA-48_ and *bla*_KPC-2_ and novel plasmid structures were identified.

## Results

### Strains characteristic

*K. pneumoniae* EBSI041 was collected from the blood sample of a male emergency ICU patient in March 2012 in Egypt. This strain showed high resistance to almost all tested antibiotics, including imipenem, meropenem, ertapenem, piperacillin-tazobactam, cefotaxime, ceftazidime, cefepime, aztreonam, gentamicin, amikacin, ciprofloxacin, fosfomycin, chloramphenicol, but sensitivity to colistin, tigecycline, tetracycline, and trimethoprim-sulfamethoxazole (Table S1). EBSI041 exhibited extensive resistance to carbapenems with high resistance levels (MIC > 32 mg/L). In addition, it was also highly resistant to piperacillin-tazobactam, a β-lactam combination agent, with MIC > 512 mg/L, and cephems cefotaxime (MIC > 256 mg/L), ceftazidime (MIC > 256 mg/L), cefepime (MIC = 256 mg/L). The virulence level of strain EBSI041 was tested in wax moth (*G. mellonella*) larvae. The larval mortality rate increased dramatically to 60% within 12 h post-infection with EBSI041. On being infected for 48 h at an inoculum of 1 × 10^4^ colony-forming units (CFU), the survival of *G. mellonella* were 10 and 0% for EBSI041 and the hypervirulence control strains HvKP4, respectively (Fig. S1).

In vitro conjugation demonstrated that carbapenem resistance genetic factors in EBSI041 can be transferred to the recipient strains *E. coli* J53 and EC600. Further S1-PFGE and PCR assay revealed OXA-48-carrying plasmid (~ 90 kb) can be self-transferred and KPC-2-producing plasmid has no transferability. However, KPC-2-producing plasmid can be transferred to the recipient strains with the help of OXA-48-carrying plasmid, and the two plasmids may be fused a larger plasmid (~ 180 kb) carring both *bla*_OXA-48_ and *bla*_KPC-2_ according to results of S1-PFGE (Fig. S2).

### Genomic features of the carbapenem-resistant hypervirulent *K. pneumoniae* EBSI041

Genomic analysis showed EBSI041 included a 5,516,355 bp chromosome and seven plasmids: pEBSI041-1 (299,522 bp), pEBSI041-2 (97,179 bp), pEBSI041-3 (85,594 bp), pEBSI041-4 (45,103 bp), pEBSI041-5 (10,060 bp, pEBSI041-6 (5596 bp), pEBSI041-7 (1780 bp) (Table 1). EBSI041 carried 19 antibiotic resistance genes (ARGs), four of them were located on the chromosomes including the *bla*_SHV-11_, *oqxB*, *oqxA*, and *fosA6* genes. The two carbapenemase genes, *bla*_OXA-48_ and *bla*_KPC-2_, were identified in plasmids pEBSI041-2 and pEBSI041-3, respectively. In addition to resistance genes, eighty-six putative virulence genes were annotated in the genome of EBSI041, including genes coding for fimbriae, capsule, yersiniabactin, iron-enterobactin, mucoid and aerobactin. Most virulence factors were found on chromosome, except for seven genes (*rmpA*, *rmpA2*, *iucABCD* and *iutA*) in plasmid pEBSI041-1. The virulence of EBSI041 was demonstrated using the *Galleria mellonella* larvae model. EBSI041 was identified as resistant and virulent ST11 clone.

**Table 1:** Overall features of *K. pneumoniae* EBSI041

Clinical characteristics		Genome characteristics					
Country	Egypt	Parameter	Size (bp)	Plasmid_type	Resistance gene	Virulence gene	Accession no.
**Unit**	ICU emergency	**Chromosome**	5,516,355	–	*oqxB* *oqxA* *bla*_SHV-11_ *fosA6*		–
**Gender**	Male						
**Type of Infection**	Septicemia						
**Sampling Site**	Blood						
**Date of Isolation**	March 2012	**pEBSI041-1**	299,522	IncHI1B:IncFIB	*armA* *msr(E)* *mph(E)* *qnrS1* *aph(3′)-VI* *sul2*	*rmpA* *iucA* *iucB* *iucC* *iucD* *iutA* *rmpA2*	MW245019
		**pEBSI041-2**	97,179	IncL/M	*bla*_OXA-48_ *bla*_SHV-12_		MW245020
		**pEBSI041-3**	85,594	IncR	*bla*_KPC-2_ *bla*_SHV-12_		MW245021
		**pEBSI041-4**	45,103	IncFII	*bla*_CTX-M-65_ *fosA3* *bla*_TEM-1B_ *rmtB* *catA2*		MW245022
		**pEBSI041-5**	10,060	ColRNAI			–
		**pEBSI041-6**	5596	ColRNAI			–
		**pEBSI041-7**	1780	ColpVC			–

For source tracking bacterial pathogens, EBSI041 was found to be similar to a ST11 *K. pneumoniae* strain (WJTB01) isolated from bronchoalveolar lavage of patient in China, with 317 SNPs, based on SNP (sequence-based) strategy using BacWGSTdb 2.0 [24]. The core-genome-based MLST (cgMLST) analysis showed that 67 strains were less than or equal to 50 alleles different from EBSI041. All strains were isolated from China, except one from USA. EBSI041 and HA_74 (PJOU01) were on the same branch of the phylogenetic tree (Fig. S3). ST11 *K. pneumoniae* strain HA_74 was isolated from China in 2015. However, because of limited sources from Egypt or Africa, we were not able to clarify the domestic transmission route.

### The MDR pEBSI041-1 co-harbouring virulence genes via recombination

**Fig. 1:**
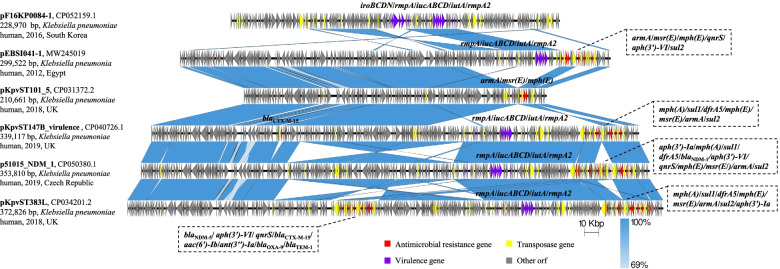
Structure analysis of pEBSI041-1. Major structural features of plasmid pEBSI041-1 were compared with plasmids pF16KP0084-1 (CP052159.1), pKpvST101_5 (CP031372.2), pKpvST147B_virulence (CP040726.1), p51015_NDM_1 (CP050380.1) and pKpvST383L (CP034201.2). Blue shading indicates shared regions with a high degree of homology. Red and purple represent the antibiotic resistance and virulence genes, respectively, and yellow is the insertion sequences and transposons

The plasmid pEBSI041-1 is 299,522 bp in size and belongs to an IncFIB:IncHI1B type hybrid plasmid. A 27,568-bp multidrug-resistance (MDR) module in pEBSI041-1 harboured six resistance genes *armA*, *msr(E)*, *mph(E), qnrS*, *aph(3′)-VI* and *sul2*. The *armA* gene was mediated by an intact IS*26* (IS*6* family, 820 bp) element upstream. With a 2300-bp space to *armA* downstream, a locus of *msr(E)* and *mph(E)* was flanked by an intact IS*Ec29* (IS*4* family, 1325 bp) (Fig. 1). For the rest of ARGs, each of them was flanked independently by different IS elements. This module was almost identical to the sequences in plasmid p51015_NDM_1 (CP050380) which was identified in a human *K. pneumoniae* isolate in the Czech Republic. The only difference between them was an inversion of a 7710-bp sequence bounded by two IS*26* occurred in p51015_NDM_1. Besides this MDR module, pEBSI041-1 harbours the plasmid-located virulence factors, including regulator of the mucoid phenotype (*rmpA*), the regulator of mucoid phenotype 2 (*rmpA2*), aerobactin (*iucABCD*, *iutA*) (Fig. 1). A BLASTn search showed that a 36,929-bp sequence containing the virulence genes in pEBSI041-1 was identical to the sequences in pF16KP0084-1 (CP052159.1; South Korea). Furthermore, close to this sequence, a 37,030-bp sequence was also found to be identical to the sequence in pF16KP0084-1 with a reversion order, while the sequence harbours the virulence gene cluster *iroBCD* and *iroN* in pF16KP0084-1 was lacking in our plasmid (Fig. 1). Also, these two sequence fragments in pEBSI041-1 caused the main difference to plasmid pKpvST101_5 (CP031372.2; United Kingdom), indicating that the emergence of the MDR-virulent pEBSI041-1 was due to the transfer of virulence determinants into a pKpvST101_5-like MDR plasmid. Sequence alignments showed that pEBSI041-1 shared > 99% identity with plasmid pKpvST147B_virulence (CP040726.1; United Kingdom), pKpvST383L (CP034201.2; United Kingdom), and p51015_NDM_1 (CP050380.1) with query coverages of 96-99.5%, all of which are MDR-virulent hybrid plasmids (Fig. 1).

### The *bla*_OXA-48_-carrying pEBSI041-2 harbours an exogenetic chromosome-located fragment

**Fig. 2:**
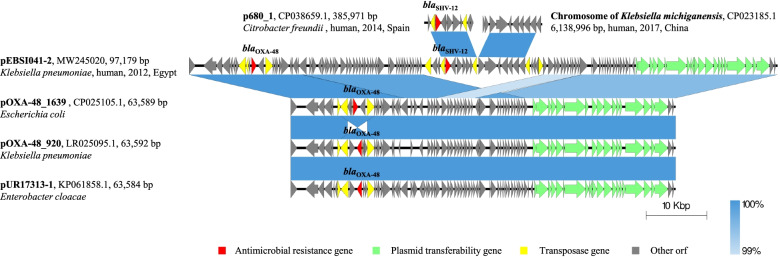
The comparative schematic diagram of plasmid pEBSI041-2. pEBSI041-2 was compared with three OXA-48-carrying plasmids pOXA-48_1639 (CP025105.1), pOXA-48_920 (LR025095.1) and pUR17313-1 (KP061858.1). Besides, plasmid p680_1 (CP038659.1) and the chromosome of *K. michiganensis* strains (CP023185.1) were analyzed. Red represent the antibiotic resistance, and yellow is the insertion sequences and transposons. Plasmid transferability genes were shown in green

The IncL/M-type plasmid pEBSI041-2 was 97,179 bp in size with an average GC content of 52.45% and contained 134 open reading frames (ORFs). It possessed a complete array of genes involved in replication (*repA* gene), stabilization (*stbAB* genes), toxin-antitoxin system (*pemIK* genes) and conjugation (*traHIJKLMNOPQR* and *traUWXY*X genes). The *bla*_OXA-48_ gene, encoding the class D carbapenemase OXA-48, was found in this plasmid. The *bla*_OXA-48_ gene was surrounded by multiple IS elements, namely ΔIS*10A* (IS*4* family, 1329 bp), IS*1R* (IS*1* family, 768 bp) and IS*10A* (IS*4* family, 1329 bp) (Fig. 2). pEBSI041-2 exhibited a high similarity (80% coverage, 99.58% identity) with plasmid pOXA-48_1639 (CP025105.1), which also encodes OXA-48 and identified from *E. coli*. Besides, pEBSI041-2 was also similar to plasmids pOXA-48_920 (LR025095.1) and pUR17313-1 (KP061858.1), which are from *K. pneumoniae* and *Enterobacter cloacae* respectively. This indicates that pOXA-48_1639-like plasmids have been widely spread among bacteria of different species. However, compared with pOXA-48_1639, an 18,779-bp sequence harbouring *bla*_SHV-12_ was missing in pEBSI036-2. The *bla*_SHV-12_ gene was surrounded by two IS*26* (IS*6* family, 820 bp) elements, and this 6100-bp fragment was similar to plasmid p680_1 (CP038659.1) from *Citrobacter freundii*. A BLASTn search did not find the homologous plasmid-located sequence to the remaining region (47,727-55,495 bp) while exhibited limited similarities (~ 50% coverage, > 99% identity) to the chromosome of some *K. michiganensis* strains (CP023185.1; CP022348.1; CP003683.1) (Fig. 2). The mobilization of *bla*_SHV-12_ can be attributed to the gene exchange by recombination, while the acquisition of exogenous chromosome sequence needs to be further considered. Indeed, the conjugation experiment demonstrated that pEBSI041-2 transferred from the donor strain *K. pneumoniae* EBSI041 to the recipients *E. coli* J53 and EC600.

### The transfer of non-conjugative *bla*_KPC-2_-carrying pEBSI041-3 needs the help of pEBSI041-2

**Fig. 3:**
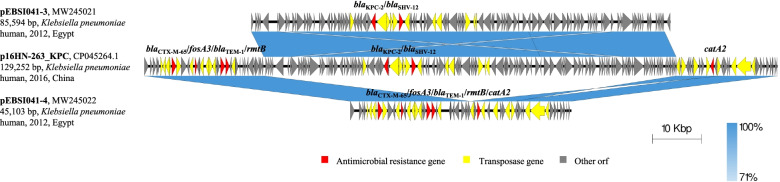
Sequence alignment analysis among plasmids pEBSI041-3, pEBSI041-4 and p16HN-263_KPC (CP045264.1). Red represent the antibiotic resistance genes, and yellow is the insertion sequences and transposons

The pEBSI041-3 was an IncR-type plasmid. In pEBSI041-3, two antibiotic resistance genes *bla*_KPC-2_ and *bla*_SHV-12_ were found, which were separated by a fragment of IS*Kpn27*-ΔTn*3*-IS*26*-ΔTn*As1* (Fig. 3). Besides, an ΔIS*Kpn6* (IS*1182* family, 1540 bp) was located in upstream of *bla*_KPC-2_. The pEBSI041-3 acquired *bla*_KPC-2_ by a transposon unit with the core structure of ΔIS*Kpn6-bla*_KPC-2_-IS*Kpn27* as pKPC-LK30 (KC405622.1) [25] and pKPC-L111 (CP030134.1) [15] and captured *bla*_SHV-12_ with an IS*26*-interrupted Tn*As1* (Tn*3* family, 6694 bp) element. The complete transfer operon (locus *tra*-*trb*) was not detected in pEBSI041-3, except *traA* and *traM*, which may explain why pEBSI041-3 does not transfer conjugatively to the recipients *E. coli* J53 and EC600. However, the results of vitro conjugation and PFGE experiments showed pEBSI041-3 can be transferred with the help of conjugative plasmid pEBSI041-2. Moreover, pEBSI041-2 and pEBSI041-3 may fuse into a larger plasmid (~ 180 kb) carried both *bla*_OXA-48_ and *bla*_KPC-2_ genes during co-transfer (Fig. S2).

### Potential recombination of plasmids pEBSI041-4 and pEBSI041-3

Plasmid pEBSI041-4 was identified as an IncFII type. A 20,894 bp multi-drug resistance region in pEBSI041-4 harboured five resistance genes, including *bla*_CTX-M-65_, *bla*_TEM-1B_, *catA2*, *fosA3* and *rmtB*. Those genes were separated by eight IS*26* elements, one IS*903B* (IS*5* family, 1057 bp) element, one IS*Kpn26* (IS*5* family, 1196 bp) element, one IS*Cfr3* (IS*NCY* family, 1081 bp) element and a few truncated insertion sequences and transposons (Fig. 3).

The segment contained genes *bla*_CTX-M-65_, *bla*_TEM-1B_, *fosA3*, and *rmtB* was similar to F33:A-:B-type plasmid pHN7A8, which isolated from an *E. coli* in China [26]. The pEBSI041-4 shared 99.98% identity with pHN7A8 (JN232517.1) with query coverages of 71%. Compared to pHN7A8, pEBSI041-4 has an additional resistance gene *catA2* flanked by two IS*26* elements. Besides, the downstream region of *catA2* has IS*Kpn26*, IS*Cfr3* and IS*26* elements. The presence of multiple insertion sequences, especially IS*26*, indicates that recombination may repeatedly have occurred in this multi-drug resistance region.

Moreover, the combination of pEBSI041-3 and pEBSI041-4 was almost identical to p16HN-263_KPC (CP045264.1) (Fig. 3). The p16HN-263_KPC was collected from *Klebsiella pneumoniae* of bloodstream infection in China and shared high similarity with pKP1034 (KP893385.1) and p69-2(CP025458.1) [27, 28]. Those KPC-2-producing plasmids belonged to IncR-F33:A-:B- type and carried all resistance genes in pEBSI041-3 and pEBSI041-4, except p69-2 without *fosA3*. The comparison suggested that pEBSI041-3 and pEBSI041-4 might have undergone rearrangement by recombination to form p16HN-263_KPC-like plasmids.

## Discussion

ST11 is the dominant clone of carbapenemases-producing *K. pneumoniae* in Asia, especially in China, and ST11 clone was reported to account for up to 60% of carbapenem-resistant *K. pneumoniae* [9, 29, 30]. Recently, ST11 has been reported in several clinical infection cases from African countries, such as Egypt [31, 32] and Tunisian [33]. And in ST11 carbapenem-resistant *K. pneumoniae* isolates, the most predominant carbapenemase genes are *bla*_KPC-2_, *bla*_NDM-1_ and *bla*_OXA-48_ [10, 34]. The emerging threat of carbapenem resistance of *K. pneumoniae* in Egyptian hospitals has been highlighted over recent years [3, 35]. To the best of our knowledge, this is the first report on the complete genome sequence of KPC-2-and OXA-48-coproducing virulent *K. pneumoniae* from Egypt.

The uncommon co-carriage of genes encoding different classes of carbapenemases endowed EBSI041 with high carbapenems resistance. Not restricted in this study, the co-harboring *bla*_KPC-2_ and *bla*_OXA-48_ in *K. pneumoniae* isolates were also found from other clinical setting [15]. The presence of carbapenemase genes on mobile elements greatly promotes the spread and stacking of carbapenems resistance. The *bla*_OXA-48_-carrying pEBSI041-2 was identified as an IncL/M-type plasmids, which are commonly self-conjugative among *Enterobacteriaceae* according to the previous studies [36]. It’s worth noting that non-conjugative pEBSI041-3 carried *bla*_KPC-2_ gene, was successfully transferred with the help of pEBSI041-2. The results warn that the mechanisms of *bla*_OXA-48_ or *bla*_KPC-2_ -carrying plasmid transfer need to be further studied to better control the spread of carbapenemase-producing *K. pneumoniae*.

The genetic structure of *bla*_OXA-48_ or *bla*_KPC-2_ -carrying plasmid in this study is different from that of reported plasmids due to the presence of multiple transposons and insertion sequences. The *bla*_OXA-48_-carrying pEBSI041-2 was similar to other IncL/M plasmids previously sequenced, the majority of which only carry *bla*_OXA-48_ [15]. However, pEBSI041-2 harboured an exogenetic chromosome-located fragment and acquired additional resistance gene *bla*_SHV-12_ due to the recombination of the IS*26*-like elements. In pEBSI041-3, the *bla*_KPC-2_ gene was located on a transposon unit with the core structure of ΔIS*Kpn6-bla*_KPC-2_-IS*Kpn27* as that reported [15, 25]. Further, pEBSI041-3 and MDR plasmid pEBSI041-4, which carried eight IS*26* elements, almost constitutes another KPC-2-producing plasmids [27, 28]. Therefore, the novel genetic structure of these plasmids are likely to be created by IS-mediated recombination.

## Conclusion

This study reported the co-carriage of distinct *bla*_KPC-2_ and *bla*_OXA-48_ plasmids in a single ST11 hypervirulent *Klebsiella pneumoniae* isolate in Egypt. The recombination and rearrangement of MDR plasmids and virulent plasmids have occurred during evolution. These results give new insights into the adaptation and evolution of *K. pneumoniae* plasmids during nosocomial infections in Egypt.

## Materials and methods

### Bacterial strain

*K. pneumoniae* EBSI041 was collected from the blood sample of a patient in Egypt. The clinical strain was initially isolated on MacConkey agar (Oxoid, UK). Species identification was determined primarily with an automated VITEK®2 AST-16 Gram-negative susceptibility card (bioMérieux, Marcy-l’Étoile, France) and confirmed by matrix-assisted laser desorption ionisation-time of flight mass spectrometry (MALDI-TOF MS). Investigation of carbapenemase production in the routine hospital laboratory procedure using the Modified Hodge Test (MHT) [37] and Carba NP test [38] showed that EBSI041 was resistant to carbapenems. We tested EBSI041 for carbapenems-resistant genes and confirmed that it carried *bla*_OXA-48_ and *bla*_KPC-2_. Then, EBSI041 was selected for whole-genome sequencing for further identification. Ethical approval for this study was given by Zhongshan School of Medicine of Sun Yat-sen University under approval number 068. All methods involved in this study were carried out in accordance with relevant guidelines and regulations.

### Antimicrobial susceptibility testing

Minimum inhibitory concentrations (MICs) were determined for the following 17 different antibiotics: cefotaxime (CTX), ceftazidime (CAZ), cefepime (FEP), colistin (CT), tigecycline (TGC), imipenem (IMP), ertapenem (ETP), meropenem (MEM), ciprofloxacin (CIP), fosfomycin (FOS), trimethoprim-sulfamethoxazole (SXT), piperacillin-tazobactam (PTZ), amikacin (AMK), gentamicin (GEN), chloramphenicol (CHL), tetracycline (TET), and aztreonam (ATM) for EBSI041 using the agar microdilution method excepted for colistin using the broth microdilution method. *Escherichia coli* ATCC 25922 strain was used as the reference control. MICs were interpreted following the Clinical and Laboratory Standards Institute (CLSI 2018) [39] guidelines, except for tigecycline and colistin, which were interpreted using the EUCAST 2019 guidelines (ECOFFs; http://www.eucast.org/).

### In vitro conjugation and S1-PFGE

The horizontal transfer of plasmids was examined by in vitro conjugation using *K. pneumoniae* EBSI041 as a donor and *E. coli* strain EC600 and J53 as a recipient, respectively. Briefly, the EBSI041, EC600 and J53 strains were cultured to OD_600_ 0.4-0.6, mixed in a 1: 1 donor-to-recipient ratio, platted onto Luria-Bertani (LB) agar plates and incubated at 37 °C overnight. One ml of sterile saline was used to remove the conjugation mix from the LB agar plates. Transconjugants were then selected by plating LB agar plates containing rifampicin (Rif; 500 μg/ml) and imipenem (Imp; 2 μg/ml) for EC600, and sodium azide (NaN3; 100 μg/ml) and imipenem (Imp; 2 μg/ml) for J53. The transfer of the plasmid was checked by PCR analysis and MICs. The *bla*_KPC-2_ and *bla*_OXA-48_ genes were confirmed by PCR and sequencing with primers KPC-A (TGTAAGTTACCGCGCTGAGG), KPC-B (CCAGACGACGGCATAGTCATF) [40], and OXA-A (TTGGTGGCATCGATTATCGG), OXA-B (GAGCACTTCTTTTGTGATGGC) [41]. And S1-PFGE was used to determine the sizes and numbers of plasmids harboured by the isolate EBSI041 and transconjugants [42].

### Galleria mellonella virulence assay

The virulence of strain EBSI041 was tested using the wax moth (*Galleria mellonella*) larvae model. Briefly, 30 larvae weighing about 300 mg were randomly selected for each isolate and maintained on woodchips in the dark at 15 °C until being used. Overnight cultures of *K. pneumoniae* strains were washed with phosphate-buffered saline (PBS) and further adjusted with PBS to concentrations of 1×10^6^ CFU/mL (10 ul for injection). Colony counts were conducted by serial dilution with final plating on LB agar. The *G. mellonella* were infected with the tested bacteria, as previously described [43]. PBS injection controls and the negative controls (receiving no injection) were used to evaluate trauma and attrition, respectively. EC600 strain was used as non-virulent control, while, HvKP4 strain was used as the hypervirulent control [18]. HvKP4, a ST11 carbapenem-resistant hypervirulent *K. pneumoniae* outbreak strain, was isolated from China. The larvae were incubated at 37 °C in the dark and observed every 12 h for 7 days. We recorded the survival rate of the *G. mellonella* over 48 h post-infection. Results were not included if greater than or equal to two larvae died in either of the control groups. All experiments were done in triplicate.

### Whole-genome sequencing (WGS)

The long-read MinION sequencer (Oxford Nanopore Technologies, Oxford, UK) was performed to sequencing the EBSI041 strain with a mean read length of 24 Kbp. De novo hybrid assembly was performed using Unicycler v0.4.3 [44]. Complete circular contigs were then corrected using Pilon v1.22 with Illumina reads (Illumina, USA). In silico multilocus sequence typing (MLST) was performed by MLST 1.8 (https://cge.cbs.dtu.dk/services/MLST/). The antibiotic resistance genes and virulence genes were identified using ABRicate version 0.5 (https://github.com/tseemann/abricate). Insertion sequence (IS) elements were determined with ISFinder (https://www-isfinder.biotoul.fr). The SNP (sequence-based) and core-genome-based MLST (cgMLST) strategies on BacWGSTdb 2.0 were used for source tracking bacterial pathogens, and the phylogenetic tree was generated and visualized by Grapetree [24, 45].

### Nucleotide accession numbers

The annotated sequences of all four plasmids have been deposited in the GenBank nucleotide sequence database under accession numbers MW245019 (pEBSI041-1), MW245020 (pEBSI041-2), MW245021 (pEBSI041-3) and MW245022 (pEBSI041-4).

## Supplementary Information

**Additional file 1: Table S1** Minimum inhibitory concentrations (MICs) of *K. pneumoniae* EBSI041 strain and transconjugants. **Figure S1** Virulence potential of strain EBSI041 as depicted in a *Galleria mellonella* infection model with an inoculum of 1 × 104 CFU. **Figure S2** The S1-PFGE map of *K. pneumoniae* EBSI041 strain and transconjugants. Transconjugant a and b were *E. coli* J53 as the recipient strains, transconjugant c and d were *E. coli* EC600 as the recipient strains. **Figure S3** The phylogenetic tree of 67 *K. pneumoniae* strains based on core-genome-based MLST (cgMLST) analysis using BacWGSTdb 2.0 (threshold 50). The tree was generated and visualized by Grapetree.

## Data Availability

The newly sequenced plasmid sequences are available in GenBank under accession numbers MW245019 (pEBSI041-1) (https://www.ncbi.nlm.nih.gov/nuccore/MW245019), MW245020 (pEBSI041-2) (https://www.ncbi.nlm.nih.gov/nuccore/MW245020), MW245021 (pEBSI041-3) (https://www.ncbi.nlm.nih.gov/nuccore/MW245021) and MW245022 (pEBSI041-4) (https://www.ncbi.nlm.nih.gov/nuccore/MW245022). The data in the present study are available from the corresponding author upon reasonable request.

## Abbreviations

CRKP: Carbapenem-resistant *Klebsiella pneumonia*
CR-hvKP: Carbapenem-resistant hypervirulent *K. pneumoniae*
ICU: Intensive Care Unit
S1-PFGE: S1 nuclease pulsed field gel electrophoresis
ARGs: Antibiotic resistance genes
MDR: Multidrug-resistance
ORFs: Open reading frames
MALDI-TOF MS: matrix-assisted laser desorption ionisation-time of flight mass spectrometry
MICs: Minimum inhibitory concentrations
WGS: Whole-genome sequencing
MLST: Multilocus sequence typing
