# Correction to: Carriage of distinct *bla*_KPC-2_ and *bla*_OXA-48_ plasmids in a single ST11 hypervirulent *Klebsiella pneumoniae* isolate in Egypt

**DOI:** 10.1186/s12864-022-08477-w

**Published:** 2022-04-06

**Authors:** Yanxian Yang, Yongqiang Yang, Mohamed Abd El-Gawad El-Sayed Ahmed, Mingyang Qin, Ruowen He, Yiping Wu, Xiaoxue Liang, Lan-Lan Zhong, Ping Chen, Baoguo Deng, Reem Mostafa Hassan, Weihong Wen, Lingqing Xu, Xubin Huang, Lin Xu, Guo-Bao Tian

**Affiliations:** 1 Department of Microbiology, Zhongshan School of Medicine, Sun Yat-sen University, 74 Zhongshan 2nd Road, Guangzhou, 510080 China; 2 Key Laboratory of Tropical Diseases Control (Sun Yat-sen University), Ministry of Education, Guangzhou, 510080 China; 3 School of Pharmaceutical Sciences (Shenzhen), Sun Yat-sen University, Guangzhou, 510006 China; 4 Department of Microbiology and Immunology, Faculty of Pharmaceutical Sciences and Drug Manufacturing, Misr University for Science and Technology, Cairo, 6th of October City, Egypt; 5 Department of Pathogen Biology, School of Basic Medical, Xinxiang Medical University, Xinxiang, 453003 China; 6 School of Laboratory Medicine, Chengdu Medical College, Chengdu, 610500 China; 7 Department of Clinical and Chemical Pathology, Faculty of Medicine, Cairo University, Cairo, Egypt; 8 Department of Clinical Laboratory, The Sixth Affiliated Hospital of Guangzhou Medical University, Qingyuan People’s Hospital, Qingyuan, 511518 China; 9 Department of Pulmonary and Critical Care Medicine, the First Affiliated Hospital, Sun Yat-sen University, Guangzhou, China; 10 Research Center for Clinical Laboratory Standard, Zhongshan School of Medicine, Sun Yat⁃sen University, Guangzhou, China; 11 School of Medicine, Xizang Minzu University, Xianyang, 712082 Shaanxi China

**Correction to: BMC Genomics 23, 20 (2022)**

**https://doi.org/10.1186/s12864-021-08214-9**

Following publication of the original article [1], it was reported that there was an error in the presentation of Lin Xu and Guo-Bao Tian’s affiliations in the PDF version of the article. Furthermore, the authors reported a typographical error in the sub-section ‘Antimicrobial susceptibility testing’. The correction is highlighted in bold.

The incorrect sentence was:

“Minimum inhibitory concentrations (MICs) were determined for the following 17 diferent antibiotics: cefotaxime (CTX), ceftazidime (CAZ), cefepime (FEP), colistin (CT), tigecycline (TGC), imipenem (IMP), ertapenem (ETP), meropenem (MEM), ciprofoxacin (CIP), fosfomycin (FOS), trimethoprim-sulfamethoxazole (SXT), piperacillin-tazobactam (PTZ), amikacin (AMK), gentamicin (GEN), chloramphenicol (CHL), tetracycline [5], and aztreonam (ATM) for EBSI041 using the agar microdilution method excepted for colistin using the broth microdilution method.”

The correct sentence is:

Minimum inhibitory concentrations (MICs) were determined for the following 17 diferent antibiotics: cefotaxime (CTX), ceftazidime (CAZ), cefepime (FEP), colistin (CT), tigecycline (TGC), imipenem (IMP), ertapenem (ETP), meropenem (MEM), ciprofoxacin (CIP), fosfomycin (FOS), trimethoprim-sulfamethoxazole (SXT), piperacillin-tazobactam (PTZ), amikacin (AMK), gentamicin (GEN), chloramphenicol (CHL), tetracycline **(TET)**, and aztreonam (ATM) for EBSI041 using the agar microdilution method excepted for colistin using the broth microdilution method.

The correct affiliations presentation is given in this Correction article and the original article [1] has been updated.
