# Supplementary material for: Carriage of distinct blaKPC-2 and blaOXA-48 plasmids in a single ST11 hypervirulent Klebsiella pneumoniae isolate in Egypt
Source: BMC Genomics. 2022 Jan 8;23:20. doi: 10.1186/s12864-021-08214-9 (PMC8742346; doi:10.1186/s12864-021-08214-9)
Supplement: Supplementary file 1 — Additional file 1: Table S1 Minimum inhibitory concentrations (MICs) of K. pneumoniae EBSI041 strain and transconjugants. Figure S1 Virulence potential of strain EBSI041 as depicted in a Galleria mellonella infection model with an inoculum of 1 × 10^4^ CFU. Figure S2 The S1-PFGE map of K. pneumoniae EBSI041 strain and transconjugants. Transconjugant a and b were E. coli J53 as the recipient strains, transconjugant c and d were E. coli EC600 as the recipient strains. Figure S3 The phylogenetic tree of 67 K. pneumoniae strains based on core-genome-based MLST (cgMLST) analysis using BacWGSTdb 2.0 (threshold 50). The tree was generated and visualized by Grapetree. [file 12864_2021_8214_MOESM1_ESM.doc]

**Supplementary material**

**Table S1** Minimum inhibitory concentrations (MICs) of *K. pneumoniae* EBSI041 strain and transconjugants

| Strains |  | EBSI041 | transconjugant a* | transconjugant b | transconjugant c | transconjugant d |
| --- | --- | --- | --- | --- | --- | --- |
| MICs (mg/L) | TGC | 1 | 0.25 | 0.25 | 0.25 | 0.25 |
|  | TET | 4 | 2 | ≤1 | ≤1 | ≤1 |
|  | CT | 0.5 | 0.25 | ≤0.125 | ≤0.125 | 0.25 |
|  | IMP | ＞32 | 8 | 2 | 16 | 32 |
|  | MEM | ＞32 | 2 | 0.5 | 4 | 4 |
|  | ETP | ＞32 | 32 | 16 | ＞32 | ＞32 |
|  | CHL | ＞128 | 8 | 2 | 4 | 8 |
|  | CIP | 64 | ≤0.03 | ≤0.03 | 0.25 | 0.25 |
|  | ATM | ＞256 | 256 | ＞256 | ＞256 | ＞256 |
|  | FEP | 256 | 8 | 4 | 32 | 16 |
|  | CAZ | ＞256 | 256 | 256 | ＞256 | 256 |
|  | GEN | ＞256 | ≤1 | ≤1 | ≤1 | ≤1 |
|  | AMK | ＞256 | ≤1 | ≤1 | ≤1 | 2 |
|  | CTX | ＞256 | 8 | 8 | 32 | 32 |
|  | FOS | ＞512 | 32 | 32 | ≤16 | ≤16 |
|  | SXT | 0.5 | ≤0.25 | ≤0.25 | ≤0.25 | ≤0.25 |
|  | PZT | ＞512 | 512 | 256 | ＞512 | 256 |

MICs were determined by agar dilution method except for colistin using the broth microdilution method. IPM, imipenem; MEM, meropenem; ETP, ertapenem; TZP, piperacillin-tazobactam; CTX, cefotaxime; CAZ, ceftazidime; FEP, cefepime; ATM, aztreonam; GEN, gentamicin; AMK, amikacin; CIP, ciprofloxacin; FOS, fosfomycin; SXT, trimethoprim-sulfamethoxazole; CHL, chloramphenicol; TET, tetracycline; TGC, tigecycline; CST, colistin.

*Transconjugant a and b are *E. coli* J53 as the recipient strains, transconjugant c and d are *E. coli* EC600 as the recipient strains.

**
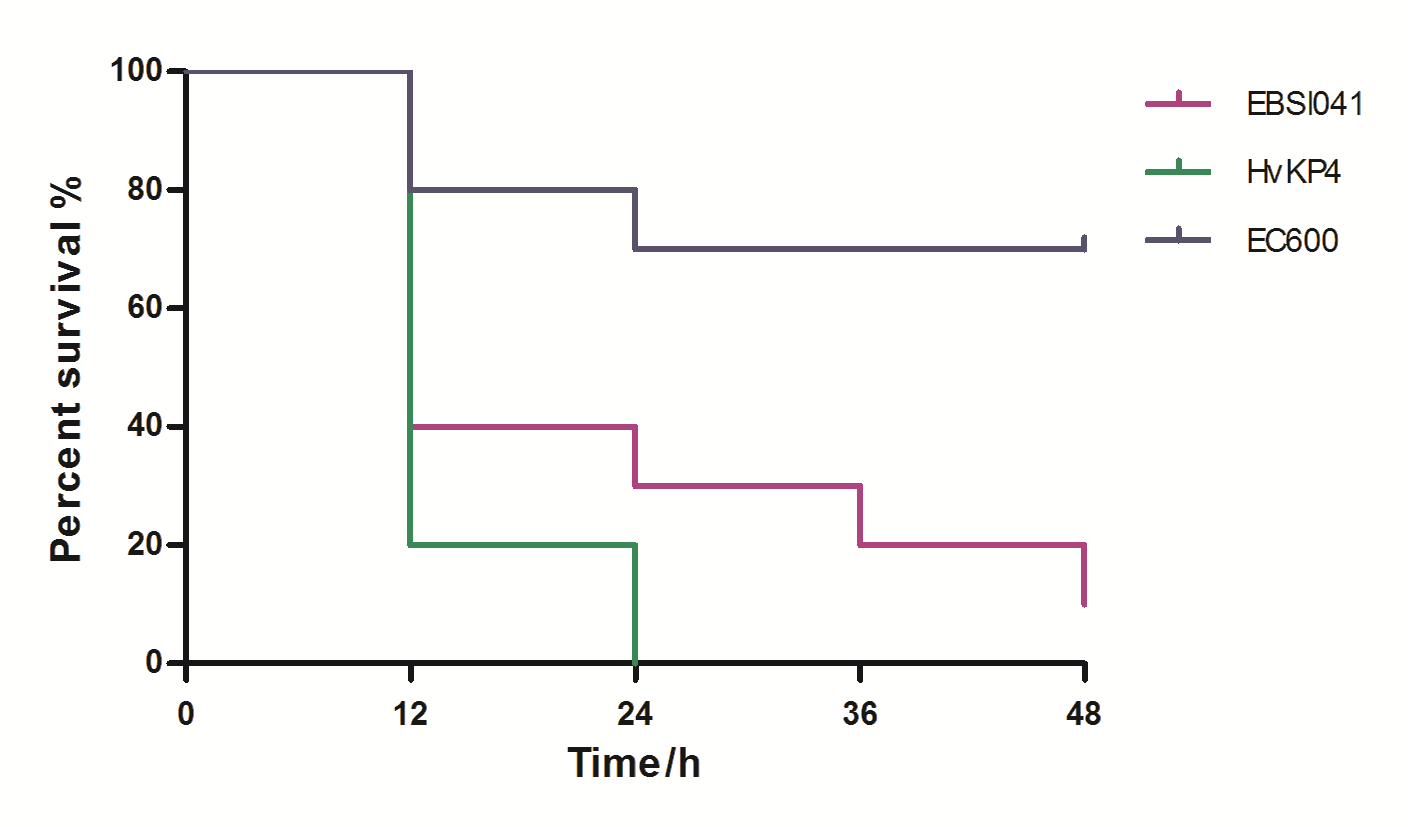
**

**Figure S1** Virulence potential of strain EBSI041 as depicted in a *Galleria mellonella* infection model with an inoculum of 1 × 10^4^CFU.


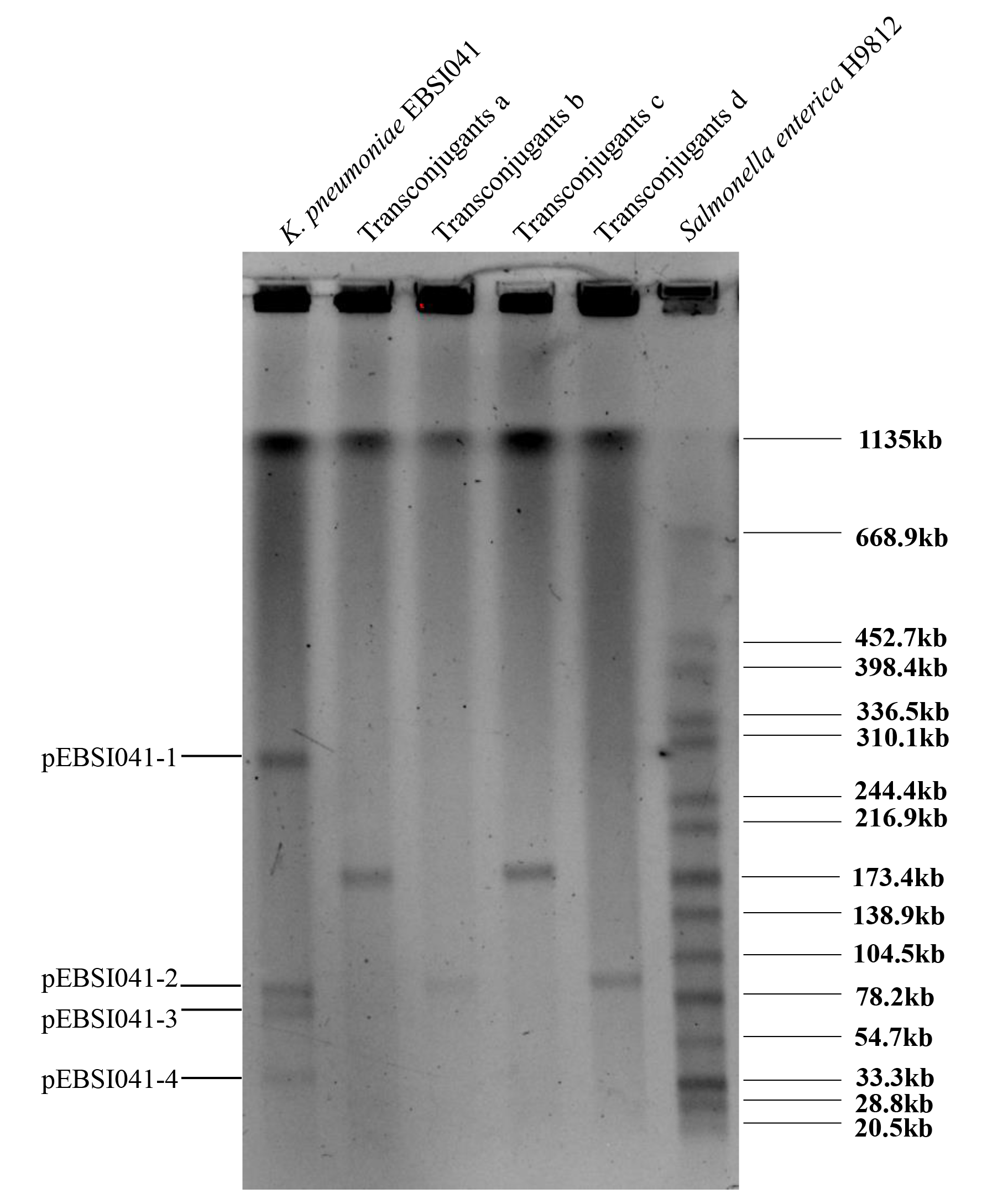


**Figure S2** The S1-PFGE map of *K. pneumoniae* EBSI041 strain and transconjugants. Transconjugant a and b were *E. coli* J53 as the recipient strains, transconjugant c and d were *E. coli* EC600 as the recipient strains.


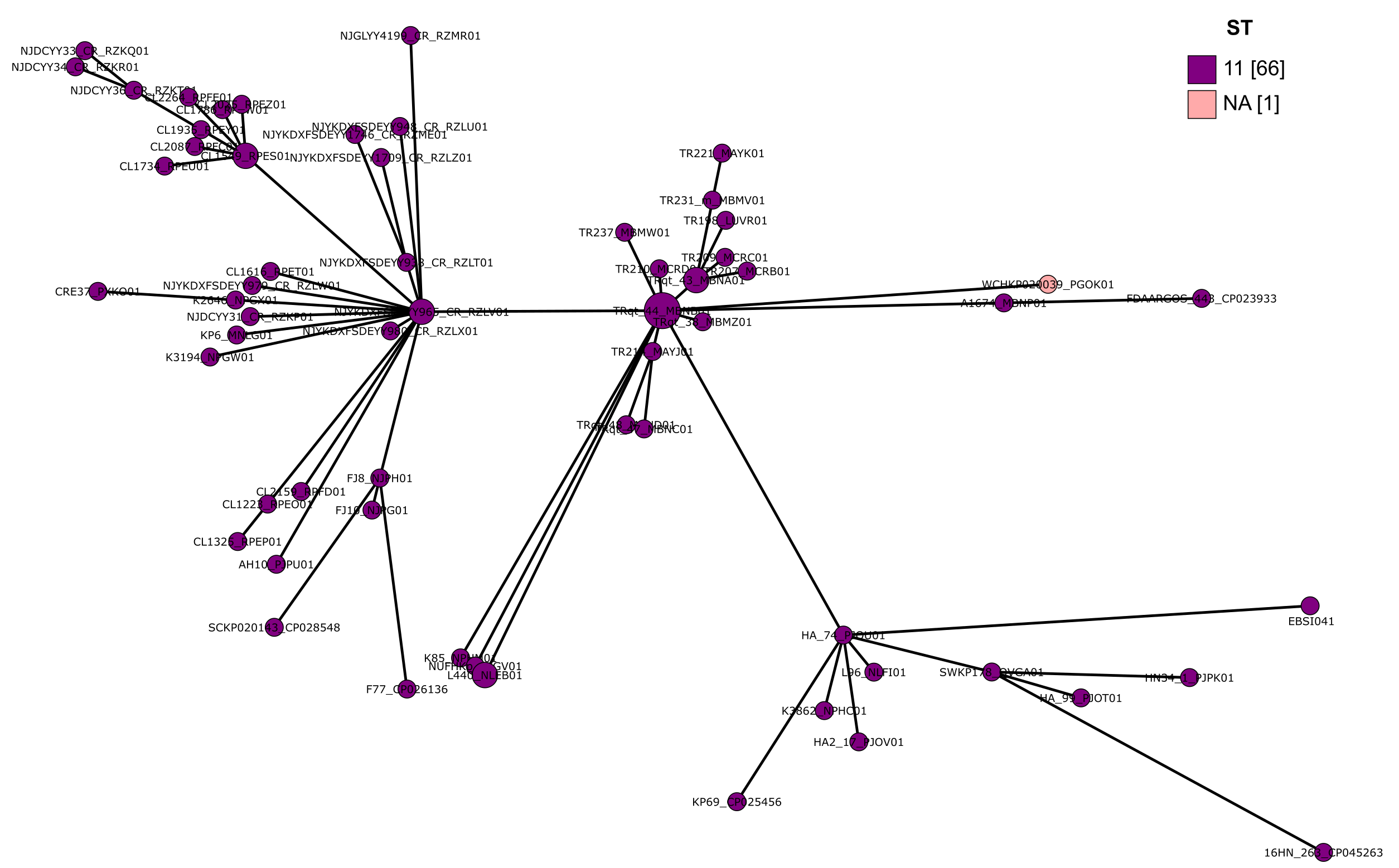


**Figure S3** The phylogenetic tree of 67 *K. pneumoniae* strains based on core-genome-based MLST (cgMLST) analysis using BacWGSTdb 2.0 (threshold 50). The tree was generated and visualized by Grapetree.
